# When the going gets tough the beautiful get going: aesthetic appeal facilitates task performance

**DOI:** 10.3758/s13423-014-0794-z

**Published:** 2015-01-17

**Authors:** Irene Reppa, Siné McDougall

**Affiliations:** 1 Department of Psychology, Swansea University, Wales, SA2 8PP UK; 2 School of Design, Engineering and Computing, Bournemouth University, Fern Barrow, Poole, BH12 5BB UK

**Keywords:** Attention, Human factors, Visual perception

## Abstract

The current studies examined the effect of aesthetic appeal on performance. According to one hypothesis, appeal would lead to overall decrements or enhancements in performance [e.g. Sonderegger & Sauer, (*Applied Ergonomics*, *41*, 403–410, 2010)]. Alternatively, appeal might influence performance only in problem situations, such as when the task is difficult [e.g. Norman, (2004)]. The predictions of these hypotheses were examined in the context of an icon search-and-localisation task. Icons were used because they are well-defined stimuli and pervasive to modern everyday life. When search was made difficult using visually complex stimuli (Experiment 1), or abstract and unfamiliar stimuli (Experiment 2), icons that were appealing were found more quickly than their unappealing counterparts. These findings show that in a low-level visual processing task, with demand characteristics related to appeal eliminated, appeal can influence performance, especially under duress.

**Electronic supplementary material:**

The online version of this article (doi:10.3758/s13423-014-0794-z) contains supplementary material, which is available to authorized users.

## Introduction

Can visual aesthetic appeal influence task performance? This is an intriguing question with potentially far-reaching practical and theoretical implications (e.g. Norman, 2004). The interest in the relationship between aesthetic appeal and performance is not new. Early studies on the relationship between aesthetic appeal and task performance revealed strong correlations between judgments of aesthetic appeal and judgments of how easy-to-use a product appears to be (e.g. Jordan, 1998; Kurosu & Kashimura, 1995; Lingaard & Dudek, 2003; Tractinsky, Katz, & Ikar, 2000; Tractinsky, 2004; Wiedenbeck, 1999; see Hassenzahl & Monk, 2010, for review). Experimental study of whether aesthetic appeal might influence actual task performance has only recently started to make headway (e.g. Thüring & Mahlke, 2007; Moshagen, Musch, & Göritz, 2009; Sauer & Sonderegger, 2009; 2011; Sonderegger & Sauer, 2010).

The handful of studies examining whether visual aesthetic appeal might influence performance has yielded mixed support for this notion. Some studies have found no effect of stimulus appeal on task performance (e.g. Hartmann, Sutcliffe, & De Angeli, 2007; Thüring & Mahlke, 2007; Tractinsky et al., 2000), while others have found conflicting results (e.g. Moshagen et al., 2009; Sonderegger & Sauer, 2009). Positive evidence suggests that appealing stimuli can increase performance efficiency (e.g. Moshagen et al., 2009; Sonderegger & Sauer, 2010) and perseverance with the task (e.g. Nakarada-Kordich & Lobb, 2005). In contrast, decreased performance efficiency for appealing stimuli has sometimes been reported (e.g. Ben-Bassat et al., 2006; Meyer, Shinar, & Leiser, 1997; Tufte, 1983; Sauer & Sonderegger, 2009; 2011).

Conflicting findings may be due to the fact that aesthetic appeal^1^ is a multi-dimensional construct influenced by a number of factors, including colour (e.g. Palmer, Schloss, & Sammartino, 2013), visual complexity (e.g., Eisenman, 1967; Jacobsen & Höfel, 2002; Martindale, Moore, & West, 1988), symmetry and balance (e.g. Jacobsen & Höfel, 2002; Palmer & Griscom, 2013), meaningfulness (e.g. Russell, 2003; Leder, Carbon, & Ripsas, 2006), familiarity (e.g. Bornstein, 1989; Lindgaard, Fernandes, Dudek, & Brown, 2006; Reber, Schwarz, & Winkielman, 2004; Zajonc, 1968, 1998, 2000), and concreteness (e.g. Kawabata & Zeki, 2004; Vartanian & Goel, 2004), to name a few. Such factors may be confounding variables underlying the conflicting evidence regarding whether aesthetic appeal influences performance. For instance, sometimes manipulations of appeal have affected the visual complexity of the artifact under investigation (e.g. computerized mobile phone: Sonderegger & Sauer, 2010; computerised phone-book: Ben-Bassat et al., 2006), potentially confounding explanations of appeal with explanations of visual complexity.

Two theoretical accounts have been proposed to explain the effects of aesthetic appeal on performance (e.g. Norman, 2004; Sonderegger & Sauer, 2010, 2011). One account is the positive affect mediation hypothesis (e.g. Norman, 2004; Moshagen et al., 2009; see also Ashby & Isen, 1999). According to this hypothesis, in problem-solving situations, aesthetic appeal will increase the observer’s positive affect, which in turn will facilitate performance. To our knowledge, only Moshagen et al. (2009) have found support for the ‘positive affect mediation hypothesis’. They orthogonally manipulated aesthetic appeal (high vs. low) and ease of use (high vs. low) of websites to examine their combined effect on a website search-for-information task. When the website was highly usable, appeal made no difference in the two performance measures. However, for websites of low usability (e.g., limited number of options and requiring expert prior knowledge), aesthetic appeal led to better task performance. However, no explicit control of possible confounding variables was undertaken in this pioneering investigation that used a semantically laden and high-level cognitive task (searching websites for specific information).

Another account of effects of appeal on performance is the prolongation of joyful experience / increased motivation hypothesis (Sonderegger & Sauer, 2010), which suggests that appeal can either enhance or produce decrements in task performance (although it is currently unclear when enhancements or decrements in performance might occur). The ‘increased motivation’ effect is reflected in increments in performance with appealing interfaces, as the user is ‘put at ease’ or ‘in the flow’ (e.g. Csıkszentmihalyi, 1997; Lindgaard, 2007) and focuses on task completion, while the ‘prolongation of joyful experience’ effect is reflected in decrements in performance, resulting if the user seeks to prolong enjoyment rather than completing the task in hand. Investigations of this hypothesis have not manipulated other possible confounding variables, nor examined performance differences as a function of differing levels of difficulty (problem-solving or otherwise). Although neither of the two aforementioned theoretical accounts are fully explanatory of effects of aesthetic appeal on performance, they provide testable predictions for the current study.

In the present experiments we examined the effect of aesthetic appeal on performance while carefully controlling for variables that are highly correlated with appeal and are known to have a significant effect on performance. To this end, we needed a micro-world of well-defined and controlled stimuli that can allow examination of the aesthetic appeal-performance relationship, while carefully controlling for confounding variables. One such stimulus micro-world is icons, whose characteristics are well documented both regarding their relationship with ratings of appeal and regarding task performance (e.g. McDougall, Curry, & de Bruijn, 1999; McDougall, de Bruijn & Curry, 2000; McDougall & Reppa, 2008). McDougall and Reppa (2008) found that, out of a large number of icon characteristics, three accounted for a significant amount of the variance in aesthetic appeal ratings. Familiarity was the best predictor of appeal ratings, with the most familiar icons also rated as the most appealing. The second best predictor was rated visual complexity (the amount of detail in the icon), with the simpler icons rated as most appealing. Finally, icon concreteness (the extent to which icons depict real objects) ratings also predicted appeal, with the most concrete icons rated as most appealing, but only if familiarity was not accounted for (familiarity subsumed any predictive power of concreteness).

Perhaps surprisingly, the same factors are known to influence performance in tasks using icons. In icon search-and-localisation tasks, familiar icons are found faster than their unfamiliar counterparts (e.g. Isherwood, McDougall, & Curry, 2007); simple icons are found faster than complex ones (e.g. Byrne, 1993; McDougall et al., 2000; McDougall & Isherwood, 2009; McDougall, Tyrer & Folkard, 2006; Scott, 1993); and concrete icons are identified faster and more accurately than abstract icons (e.g. McDougall et al., 2000; Green & Barnard, 1990; Rogers & Oborne, 1987; Stotts, 1998).

In sum, visual complexity, concreteness, and familiarity contribute to (e.g., Jacobsen & Höfel, 2002; Martindale et al., 1988; Kawabata & Zeki, 2004; Vartanian & Goel, 2004; Zajonc, 1968, 1998, 2000), while also being strongly correlated with (e.g. McDougall & Reppa, 2008), ratings of aesthetic appeal while at the same time having been shown to affect performance (e.g. Byrne, 1993; Green & Barnard, 1990; Isherwood et al., 2007; McDougall et al., 2000; McDougall & Isherwood, 2009; McDougall et al., 2006; Rogers & Oborne, 1987; Scott, 1993; Stotts, 1998). Therefore, any examination of performance with appealing stimuli will need to control such stimulus factors, to ensure that any effects of appeal do not actually reflect effects of factors contributing to appeal and performance.

## Current Experiments

In two experiments we examined whether visual aesthetic appeal could affect the efficiency with which users find icons on displays. In a search-and-localisation task with a fixed number of distractors, participants first memorised a target icon and then searched for it among an array of nine icons. This task is designed to be analogous to the kind of task users face during interaction with interfaces where they need to find icons that match something that they wish to do (see Böcker, 1993, p. 76, for a discussion of this type of task; see also McDougall et al., 2000; Isherwood et al., 2007 for the use of this paradigm).^2^ Critically, icon visual complexity, concreteness and familiarity (dimensions known to affect performance in icon localisation tasks) were controlled to assess whether any influences of aesthetic appeal on performance could be observed independently of these contributing dimensions. In order to prevent demand characteristics relating to appeal from influencing task performance, appeal for the icons used here was pre-experimentally determined using independent ratings (see McDougall and Reppa, 2008). Appeal ratings obtained after task completion would have been contaminated from familiarity with the icons and were not collected.

Aesthetic appeal could have an independent influence on task performance over and above factors that are highly correlated with it. Some evidence that this might be the case comes from studies showing efficient orienting to higher-order features, such as emotion or threat (e.g. Becker, Anderson, Mortensen, Neufield, & Neel, 2011; Ohman, Flykt, & Estevs, 2001; Eastwood, Smilek, & Merikle, 2001; Fox, Russo, Bowles, & Dutton, 2001; LeDoux, 1996; but see Fox, Lester, Russo, Bowles, Pichler, & Dutton, 2000; Nothdurft, 1993; Purcell, Stewart, & Skov, 1996), novelty (e.g., Jonides & Yantis, 1988; Yantis & Jonides, 1984; see Wolfe, 2001, for discussion), or physical attractiveness (e.g., Maner, Kenrick, et al., 2003). Aesthetic appeal may similarly have a ubiquitously biasing effect on perception and performance, which could be either negative (with appealing icons resulting in a slower response consistent with the ‘prolongation of joyful experience’) or positive (with appealing icons resulting in a faster response due to ‘increased motivation’; Sonderegger & Sauer, 2010).

Alternatively, aesthetic appeal might be expected to interact with stimulus complexity, concreteness, or familiarity, providing a booster effect on performance (e.g. Norman, 2004) as predicted by the ‘positive affect mediation’ hypothesis. Previous evidence has shown that appeal can improve performance in problematic situations, where positive affect may help overcome obstacles in performance (e.g. Moshagen et al., 2009; see also Tractinsky et al., 2000 for a similar suggestion). This could predict that in the current studies icon localization performance would be more efficient for aesthetically pleasing hard-to-find icons, but yield no benefit for easy-to-find icons.

## Experiment 1

Experiment 1 examined the influence of aesthetic appeal and visual complexity on performance in an icon search-and-localisation task. Participants carried out an icon search-and-localisation task with exposure to the icons over nine trial blocks, making it possible to also examine the longevity of the effects of visual complexity and appeal. Although it is known that visual complexity remains detrimental to performance even after extensive experience with icons (e.g. McDougall et al., 2000), the longevity of the effects of appeal on performance have not been examined.

## Method

### Participants

Nineteen undergraduate and postgraduate Swansea University students (three males) aged between 19 and 24 years old (M = 21.2, SD = 1.3), with normal or corrected to normal vision took part in Experiment 1, in exchange for course credit. Participants were naïve to purpose of the study.

## Materials

Stimuli were icons presented in their original grey-scale form. The icons had been rated previously (McDougall et al., 1999) for complexity, concreteness, and familiarity using Likert scales ranging from 1 to 5 for each of the dimensions (Table 1). Complexity referred to the amount of detail or intricacy in the icon (1 = very simple, and 5 = very complex). Concreteness referred to the extent that images in the icons depicted something that can be found in real life (1 = definitely abstract, 5 = definitely concrete). Familiarity referred to perceived familiarity of the icon (1 = very unfamiliar, 5 = very familiar). Ratings for each icon characteristic were obtained from separate groups of participants (see McDougall et al., 1999 for details).

**Table 1:** Mean ratings (and standard deviations) of icon aesthetic appeal, visual complexity, concreteness and familiarity in each experimental condition and the results of one-way analyses and Newman-Keuls comparisons examining differences between icon ratings in each condition in Experiment 1. The Appeal values and statistics are from McDougall & Reppa (2008), and the Complexity, Concreteness, and Familiarity values are from McDougall et al. (1999). All ratings were on a 1–5 scale, with 5 representing a high value of the characteristic concerned. The symbols ‘>’ and ‘<’ mean higher and lower ratings respectively, while the ‘=’ symbol means no difference in the rated dimension. *AC* Appealing complex, *AS* appealing simple, *UC* unappealing complex, *US* unappealing simple

	Icon type				Results of statistical analyses	
Icon characteristics	AC	AS	UC	US	*F*-value	Newman-Keuls comparisons
Appeal	3.50 (0.10)	3.49 (0.53)	2.45 (0.15)	2.61 (0.10)	F(3,36) = 40.03, *P* < .001	AC = AS > UC = US
Complexity	3.49 (0.15)	1.68 (0.80)	3.69 (0.26)	1.82 (0.23)	F(3,36) = 48.48, *P* < .001	AC = UC > AS = US
Concreteness	3.85 (1.11)	3.61 (0.88)	3.26 (0.90)	2.27 (0.84)	F < 1, *P* > .05	AC = AS = UC = US
Familiarity	3.19 (0.62)	3.59 (0.94)	2.68 (0.87)	2.96 (0.85)	F(3,36) = 2.17, *P* > .05	AC = AS = UC = US

**Fig. 1:**
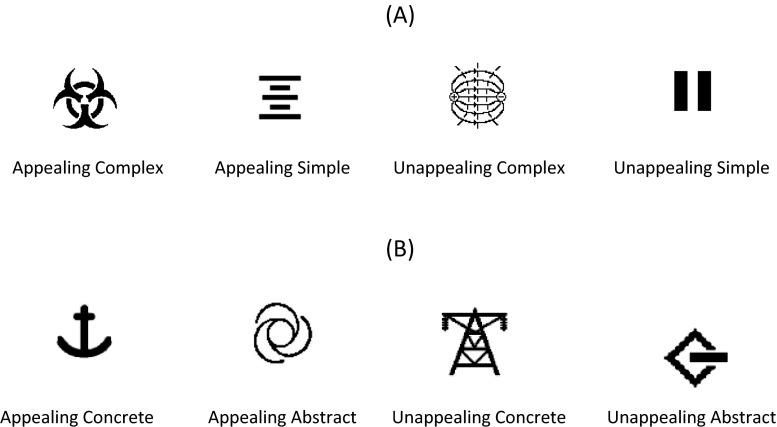
**a** Examples of icons used in Experiments 1 and 3. **b** Examples of icons used in Experiments 2 and 4

Appeal ratings for the same icon corpus appear in Table 1. Appeal ratings had been obtained by a different group of participants and reported in McDougall and Reppa (2008). They were obtained using a 5-point Likert-scale (e.g. “How much do you like this icon?”) with 1 corresponding to “really dislike” and 5 to “really like”. Forty icons that allowed the orthogonal control of rated appeal and rated visual complexity were selected from the icon corpus, leading to four unique Icon Types: appealing complex, appealing simple, unappealing complex, and unappealing simple (Fig. 1). The four Icon Types were matched in terms of Concreteness and Familiarity (see Table 1 for details).

### Design

A 2 (Complexity: complex vs. simple) × 2 (Appeal: appealing vs. unappealing) × 9 (Block: 1–9) repeated measures design, yielded 36 within-participants conditions was used. The combination of icon Complexity and Appeal yielded four icon types. There were ten unique icons of each Icon Type, for a total of 360 trials (40 trials in each of 9 blocks). Each icon was presented once as a target and eight times as a distractor per block. The dependent measure was response time (RT).

### Procedure

**Fig. 2:**
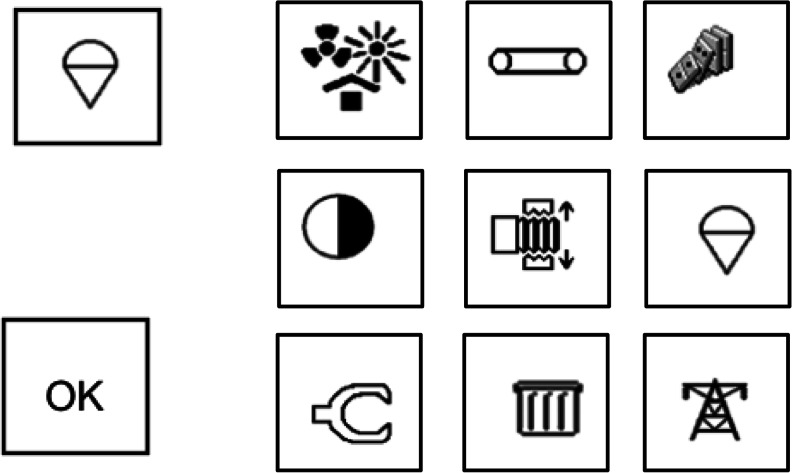
Example of an experimental trial (see Procedure for details). Placeholders were visible throughout the trial

To start each trial, participants used the mouse to click an “OK” button on the bottom left corner of the computer screen (Fig. 2). The target icon was then presented alone for 2 s at the top left corner of the screen. Following target offset participants clicked once again on the “OK” button to trigger presentation of the 9-icon array. This ensured that participants started each trial with the mouse pointer at the same point on the display. Participants had to click on the target icon as quickly as possible. The same process was repeated for 360 trials, with each icon shown nine times, once in each position in the array. Incorrect responses received a 500-ms beep sound.

## Results

Error trials (1.04 %) and trials with search RT greater or equal to 3 s (0.04 %) were excluded from the response time analysis. There was no difference in errors between any of the four icon types (all P values > .05). For both experiments reported here we used an alpha level of .05 for all statistical tests and epsilon squared (ε^2^) as an unbiased measure of effect size (e.g. Jaccard, 1998).

**Fig. 3:**
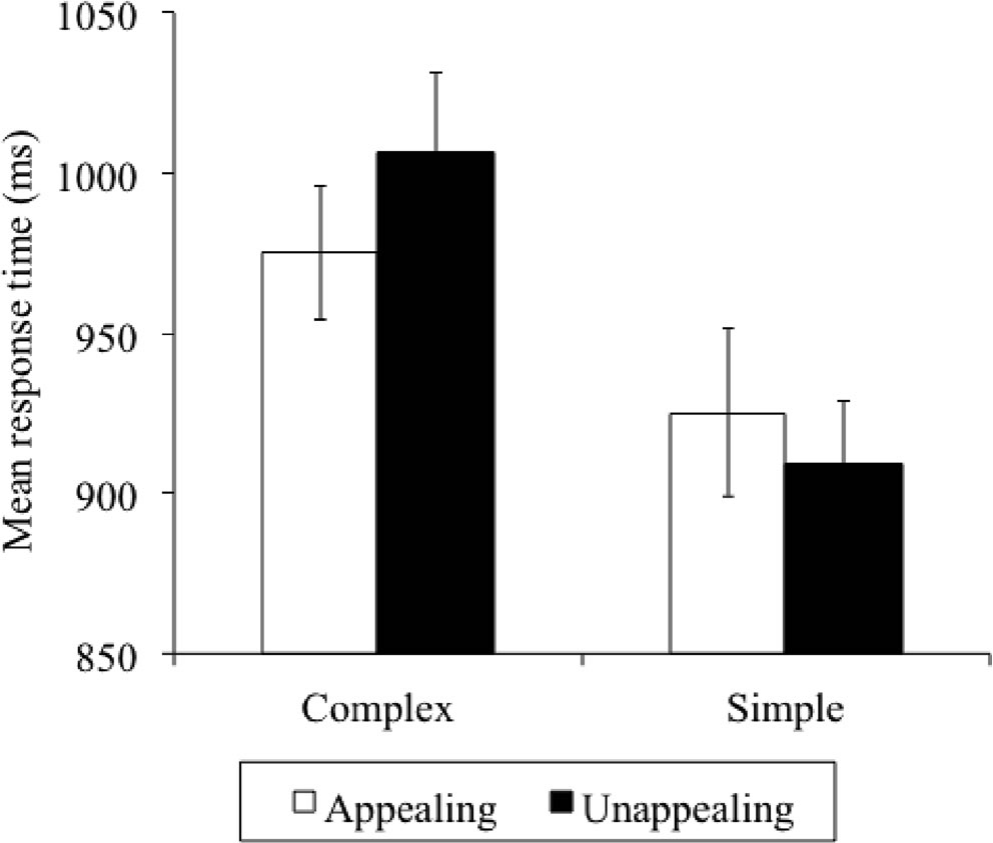
Illustration of the Complexity × Appeal interaction in Experiment 1. *Error bars* Standard error of the mean

Correct RT per condition is shown in Table 2. A 2 (Complexity: complex vs. simple) × 2 (Appeal: appealing vs. unappealing) × 9 (Block: 1–9) repeated-measures ANOVA carried out on correct RT showed no significant three-way interaction, F (8, 144) = .91, *P* > .05, ε^2^ = .04. The main effect of Complexity was significant, F (1, 18) = 94.75, *P* < .001, ε^2^ = .83, with simple icons found faster than complex icons. The main effect of Appeal was not significant, F (1, 18) = 1.04, *P* > .05, ε^2^ = .02, but there was a significant Complexity × Appeal interaction, F (1, 18) = 8.50, *P* < .01, ε^2^ = .28. Pairwise comparisons were used to examine the Complexity × Appeal interaction (see Fig. 3). To this and all other comparisons reported here a Bonferroni correction was applied. For simple icons, there was no RT difference between appealing and unappealing icons, t(18) = 1.30, *P* > .05. In contrast, complex icons were found faster if they were appealing than if they were unappealing, t(18) = 2.60, *P* = .02. Furthermore, participants found simple icons faster than complex icons, regardless of whether they were appealing t(18) = 4.95, *P* < .001, or unappealing, t(18) = 7.90, *P* < .001. Finally, there was a significant main effect of Block, F (8, 144) = 6.14, *P* < .001, ε^2^ = .25, showing that RT reduced as participants gained experience. Neither the Block × Appeal, not the Block × Complexity interactions were significant [F (8, 144) = 1.64, *P* > .05, ε^2^ = .08; F (8, 144) = .54, *P* > .05, ε^2^ = .02, respectively].

**Table 2:** Mean response times (and standard deviations) in milliseconds per Complexity and Appeal condition in Experiment 1 across the nine blocks of trials

	Icon type				
Block of trials	AC	AS	UC	US	Total
Block 1	1,072.5 (174.75)	1,044.7 (167.96)	1,058.0 (152.60)	941.5 (102.71)	1,029.2 (52.56)
Block 2	973.2 (118.63)	952.9 (137.23)	1,045.8 (135.23)	926.9 (140.24)	974.7 (51.05)
Block 3	993.1 (170.73)	949.4 (155.60)	1,002.6 (155.38)	925.0 (134.17)	967.5 (32.68)
Block 4	962.2 (75.06)	906.8 (121.09)	1,000.1 (154.14)	924.9 (165.51)	948.5 (40.44)
Block 5	951.8 (119.44)	909.6 (132.90)	1,051.4 (158.97)	892.6 (99.34)	951.3 (71.18)
Block 6	985.4 (159.73)	884.4 (122.11)	1,022.2 (184.54)	924.2 (140.43)	954.1 (58.15)
Block 7	957.1 (170.48)	908.1 (168.80)	984.6 (147.26)	894.2 (136.94)	936.0 (39.97)
Block 8	960.3 (180.35)	882.8 (165.51)	971.7 (137.55)	883.2 (129.85)	924.5 (42.23)
Block 9	921.0 (101.15)	888.6 (133.47)	924.1 (158.76)	870.7 (109.47)	901.1 (22.41)
Total	975.2 (141.1)	925.3 (145.0)	1,006.7 (153.8)	909.3 (128.7)

## Discussion

Replicating previous findings, visual complexity influenced icon localisation times, with simple icons found faster than complex icons overall (e.g. Byrne, 1993; McDougall et al., 2000; 2006; Scott, 1993). As with previous evidence (e.g. McDougall et al., 2000; Isherwood, McDougall, & Curry, 2007), the effect of visual complexity remained significant throughout the experiment (as evidenced by the lack of Complexity × Block interaction).

Most importantly, Experiment 1 showed that aesthetic appeal influenced performance over and above visual complexity of the icons. Appealing complex icons were found faster than their unappealing counterparts, whilst simple icons were equally fast regardless of aesthetic appeal. This finding does not support accounts proposing an all-or-nothing biasing effect of appeal on performance, as predicted by the ‘prolongation of joyful experience/increased motivation’ hypothesis (e.g. Sonderegger & Sauer, 2010, 2011). When icons were simple, localisation times were shortest regardless of aesthetic appeal of the stimuli demonstrating that participants were not attempting to prolong their experience. Similarly it was not the case that motivation was increased when looking for aesthetically appealing stimuli because there were no benefits of appeal for simple icons.

Instead, the observed icon appeal by icon complexity interaction suggests that appeal has a performance boosting effect, supporting the predictions of the ‘positive affect mediation’ hypothesis (e.g. Norman, 2004; see also Moshagen et al., 2009). Aesthetic appeal facilitated performance efficiency only under duress: when the target icon was complex and thus harder to locate among distractor icons.

## Experiment 2

In Experiment 1, complex target icons created difficult task conditions and it was under these conditions that appeal appeared to enhance task performance. Another way to create a difficult performance conditions is to present abstract versus concrete target icons; abstract icons are particularly difficult to find in arrays, especially during initial interactions with the icons (e.g. McDougall et al., 2000; Green & Barnard, 1990; Rogers & Oborne, 1987; Stotts, 1998). In Experiment 2, participants were asked to localise target icons that were matched in terms of visual complexity but varied orthogonally in rated concreteness and appeal.

## Method

### Participants

Twenty undergraduate Bournemouth University students (two males), naïve to the purpose of the experiment, aged between 20 and 23 years old (M = 20.8, SD = 0.89), and with normal or corrected to normal vision participated in exchange for course credit.

### Apparatus and materials

Forty icons were selected from the same icon corpus as Experiment 1, in a manner that orthogonally varied their rated Concreteness and Appeal, leading to four icon types: appealing concrete, appealing abstract, unappealing concrete, and unappealing abstract (see Table 3). Appeal was determined by the appeal ratings obtained by McDougall and Reppa (2008), and rated Visual Complexity, Concreteness and Familiarity were obtained from McDougall et al (1999). A set of univariate ANOVAs showed differences between the Icon Types in terms of rated Concreteness, Familiarity, and Appeal, and the lack of difference in terms of Visual Complexity (see Table 3 for details).

**Table 3:** Mean ratings (and standard deviations) for icon concreteness, aesthetic appeal, visual complexity and familiarity for each type of icon presented in Experiment 2. The Appeal values and statistics are from McDougall & Reppa (2008), and the Complexity, Concreteness, and Familiarity values are from McDougall et al., (1999). The symbols ‘>’ and ‘<’ mean higher and lower ratings respectively, while the ‘=’ symbol means no difference in the rated dimension. *AA* apealing abstract, *AC* appealing concrete, *UA* unappealing abstract, *UC* unappealing concrete

	Icon type				Results of statistical analyses	
Icon characteristics	AA	AC	UA	UC	*F*-value	Newman-Keuls comparisons
Appeal	3.52 (.18)	3.5 (.10)	2.51 (.11)	2.58 (.17)	F (3, 36) = 199.77, *P* < .001	AA = AC > UA = UC
Complexity	3.00 (.53)	2.67 (.95)	3.1 (.75)	3.22 (.76)	F (3, 36) = 1.37, *P* > .05	AA = AC = UA = UC
Concreteness	2.06 (.35)	4.57 (.25)	2.17 (.20)	4.55 (.15)	F (3, 36) = 278.48, *P* < .001	AA = UA < AC = UC
Familiarity	2.47 (.96)	3.7 (.43)	2.08 (.57)	3.78 (.35)	F (3, 36) = 18.89, *P* < .001	AA = UA < AC = UC

As shown in Table 3, the four icons types differed not only in terms of Concreteness but also in terms of Familiarity. This was unavoidable because of the very high correlation between Concreteness and Familiarity ratings for the icons in the corpus (.78). Therefore, two sets of analyses of RT were carried out, one where icons were coded in terms of Concreteness and Appeal (with ten icons per condition), and another with the icons coded in terms of Familiarity and Appeal. When the icons were coded in terms of Familiarity and Appeal, there were 13 icons for familiar appealing, 10 icons for familiar unappealing, 7 icons for unfamiliar appealing, and 10 icons for unfamiliar unappealing.

### Design and procedure

The experiment was based on a 2 (Appeal: appealing vs. unappealing) × 2 (Concreteness: concrete vs. abstract) × 9 (Block: 1–9) repeated-measures design. As icons differed unavoidably in familiarity, the second design of Experiment 2 was a 2 (Appeal: appealing vs. unappealing) × 2 (Familiarity: familiar vs. unfamiliar) × 9 (Block: 1–9) within-participants design (see also Apparatus and materials). All the other aspects of the design, and the procedure were identical to Experiment 1.

## Results and discussion

**Fig. 4:**
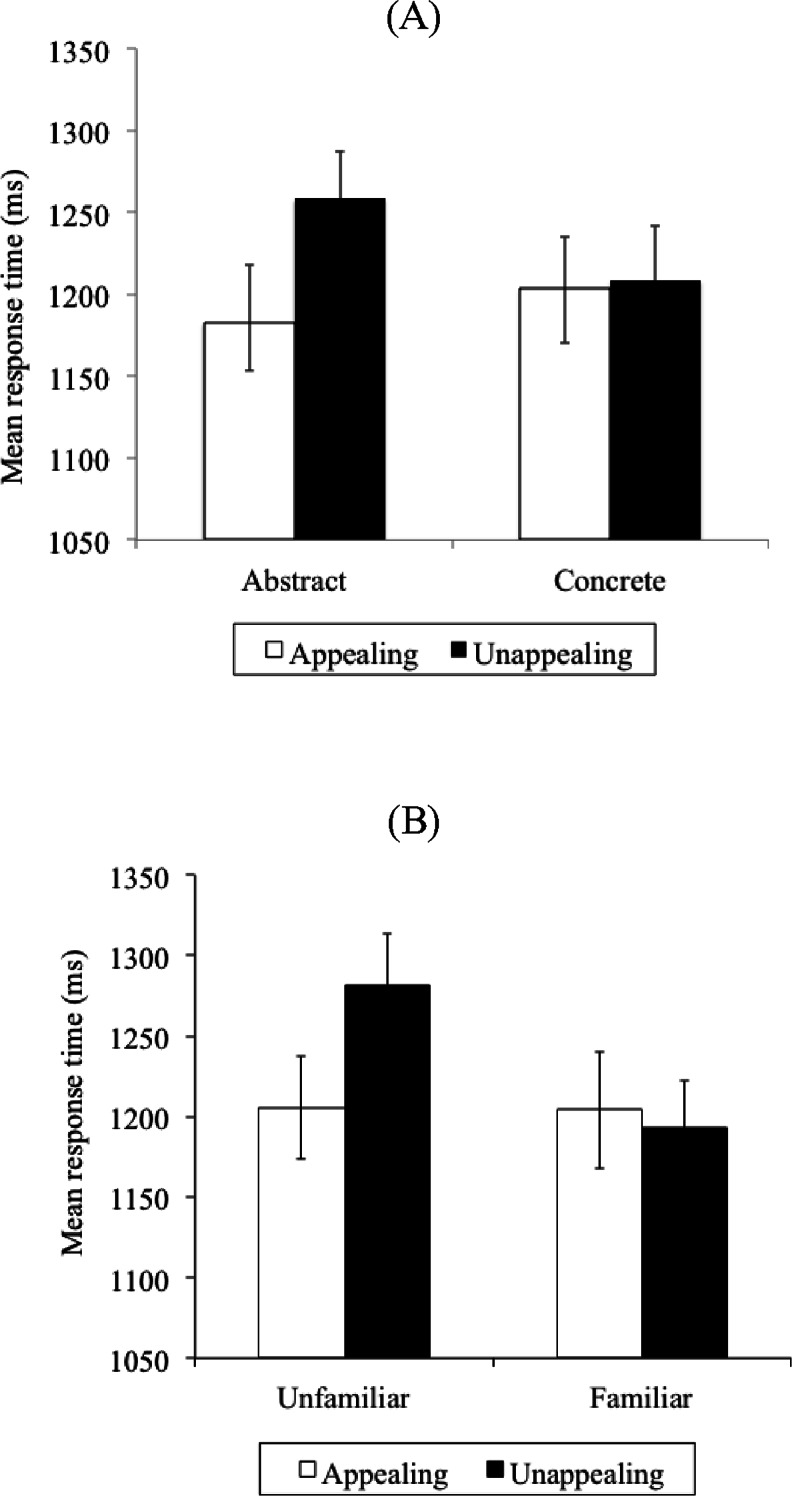
Illustrating **a** Concreteness × Appeal and **b** Familiarity × Appeal interactions in Experiment 2. *Error bars* Standard error of the mean

Errors accounted for 1.50 % of all trials. There was no difference in errors between any of the four conditions (all *P* values > .05). Trials with RT greater than 3 s accounted for 1.1 % of all correct trials and were excluded from the analysis as outliers. Correct cell means are shown in Table 4. A 2 (Concreteness: abstract vs. concrete) × 2 (Appeal: appealing vs. unappealing) × 9 (Block: 1–9) repeated-measures ANOVA on correct RT showed no significant three-way interaction, F (8,152) = 1.69, *P* > .05, ε^2^ = .08. The main effect of Appeal was significant, F (1,19) = 17.14, *P* < .001, ε^2^ = .45, with appealing icons found faster than unappealing icons. Importantly, the Concreteness × Appeal interaction was significant, F (1,19) = 14.57, *P* < .001, ε^2^ = .40 (see Fig. 4a), with shorter RT for appealing than unappealing abstract icons, t(19) = 5.50, *P* < .001, while no such differences were apparent for concrete icons, t(19) = .41, *P* > .05. Furthermore, appealing abstract icons showed no difference from appealing concrete icons [abstract appealing vs. concrete appealing: t(19) = 1.77, *P* > .05, while unappealing abstract icons took significantly longer to be localised [abstract unappealing vs. concrete unappealing: t(19) = 3.66, *P* = .002]. Thus, aesthetically enhancing abstract icons led to a comparable performance to concrete icons, while there is a significant performance cost in localising unappealing abstract icons.

**Table 4:** Mean response time (and standard deviations) per Concreteness and Appeal condition, across the nine blocks of trials in Experiment 2

	Icon type				
Block of trials	AA	AC	UA	UC	Total
Block 1	1,367.0 (252.64)	1,363.4 (171.66)	1,458.2 (242.77)	1,341.2 (243.86)	1,382.5 (44.81)
Block 2	1,271.5 (206.25)	1,204.1 (110.29)	1,260.3 (150.16)	1,206.7 (199.52)	1,235.7 (30.55)
Block 3	1,172.2 (212.23)	1,233.6 (193.31)	1,244.6 (195.82)	1,206.4 (193.68)	1,214.2 (27.96)
Block 4	1,183.3 (149.79)	1,156.6 (184.98)	1,198.0 (195.13)	1,188.5 (179.75)	1,181.6 (15.36)
Block 5	1,145.1 (185.52)	1,156.6 (168.55)	1,214.9 (174.08)	1,181.9 (196.68)	1,174.7 (26.83)
Block 6	1,211.4 (186.72)	1,166.6 (225.10)	1,271.1 (145.15)	1,201.5 (188.40)	1,212.6 (37.61)
Block 7	1,088.9 (171.09)	1,196.8 (174.00)	1,238.0 (198.15)	1,250.1 (205.28)	1,193.5 (63.54)
Block 8	1,099.5 (165.74)	1,154.9 (193.43)	1,204.5 (155.11)	1,151.2 (167.61)	1,152.5 (37.14)
Block 9	1,104.2 (199.89)	1,197.2 (154.03)	1,235.9 (180.99)	1,149.7 (167.30)	1,171.7 (49.53)
Total	1,182.6 (192.21)	1,203.3 (175.04)	1,258.4 (181.93)	1,208.6 (193.56)

While the main effect of Concreteness was not significant, F (1,19) = 2.80, *P* > .05, ε^2^ = .08, there was a significant Block × Concreteness interaction, F (8,152) = 2.75, *P* < .01, ε^2^ = .12, caused by shorter RT for concrete than abstract icons in earlier blocks of trials. Concrete targets were localised more quickly than abstract targets in Blocks 1 and 2 [t(19) = 2.28, *P* = .03; and t(19) = 2.81, *P* < .01, respectively], and the opposite relation in Block 7, t(19) = 2.48, *P* = .02. Finally, there was a significant main effect of Block, F (8,152) = 13.09, *P* < .001, ε^2^ = .40, suggesting that participants became faster with increasing experience with the icons. The Block × Appeal interaction was not significant, F (8,152) = 1.36, *P* > .05, ε^2^ = .06.

A second set of analyses was carried out, identical to the one above, but now the icons were re-coded in terms of Familiarity and Appeal. Correct cell mean RT appear in Table 5. A 2 (Familiarity: familiar vs. unfamiliar) × 2 (Appeal: appealing vs. unappealing) × 9 (Block: 1 9) repeated-measures ANOVA showed no significant three-way interaction, F (8,152) = 1.63, *P* > .05, ε^2^ = .08. All three main effects were significant: Familiarity, F (1,19) = 26.64, *P* < .001, ε^2^ = .56; Appeal, F (1,19) = 9.95, *P* < .01, ε^2^ = .31; Block, F (8, 152) = 11.45, *P* < .01, ε^2^ = .37. Critically, the Familiarity × Appeal interaction was significant, F (1,19) = 19.01, *P* < .001, ε^2^ = .47. This was the result of RT for unfamiliar icons being significantly shorter if they were appealing than when they were unappealing, t(19) = 4.56, *P* < .001, but there was no such difference for familiar icons, t(19) = .950, *P* > .05.^3^ Furthermore, there was no significant difference in RT between appealing familiar and appealing unfamiliar icons, t(19) = .14, *P* > .05, suggesting that aesthetically enhancing an unfamiliar icon causes it to behave like a familiar icon. Meanwhile, unappealing unfamiliar icons were localized significantly slower than their familiar counterparts, t(19) = 5.93, P < .0001.

**Table 5:** Mean response time (and standard deviations) per Familiarity and Appeal condition, across the nine blocks of trials in Experiment 2

	Icon type				
Block of trials	AA	AC	UA	UC	Total
Block 1	1,367.0 (252.64)	1,363.4 (171.66)	1,458.2 (242.77)	1,341.2 (243.86)	1,382.5 (44.81)
Block 2	1,271.5 (206.25)	1,204.1 (110.29)	1,260.3 (150.16)	1,206.7 (199.52)	1,235.7 (30.55)
Block 3	1,172.2 (212.23)	1,233.6 (193.31)	1,244.6 (195.82)	1,206.4 (193.68)	1,214.2 (27.96)
Block 4	1,183.3 (149.79)	1,156.6 (184.98)	1,198.0 (195.13)	1,188.5 (179.75)	1,181.6 (15.36)
Block 5	1,145.1 (185.52)	1,156.6 (168.55)	1,214.9 (174.08)	1,181.9 (196.68)	1,174.7 (26.83)
Block 6	1,211.4 (186.72)	1,166.6 (225.10)	1,271.1 (145.15)	1,201.5 (188.40)	1,212.6 (37.61)
Block 7	1,088.9 (171.09)	1,196.8 (174.00)	1,238.0 (198.15)	1,250.1 (205.28)	1,193.5 (63.54)
Block 8	1,099.5 (165.74)	1,154.9 (193.43)	1,204.5 (155.11)	1,151.2 (167.61)	1,152.5 (37.14)
Block 9	1,104.2 (199.89)	1,197.2 (154.03)	1,235.9 (180.99)	1,149.7 (167.30)	1,171.7 (49.53)
Total	1,182.6 (192.21)	1,203.3 (175.04)	1,258.4 (181.93)	1,208.6 (193.56)	

There was a significant Familiarity × Block interaction, F (8,152) = 2.47, p < .01, ε^2^ = .10, with shorter RT for familiar than unfamiliar target icons in Blocks 2 and 6 [t(19) = 3.22, *P* < .005; and t(19) = 4.72, *P* < .001, respectively]. The Appeal × Block interaction was not significant, F (8,152) = 1.9, *P* > .05, ε^2^ = .08.

Corroborating previous evidence, familiarity and concreteness influenced task performance especially in early blocks of trials (e.g. Isherwood et al., 2007; McDougall & Isherwood, 2009). This finding confirms that icon concreteness and familiarity are important variables in localization tasks and further justifies their manipulation alongside aesthetic appeal in the current study. Importantly, as in Experiment 1, icon appeal benefited performance when it was under duress—for abstract or for unfamiliar icons—but yielded no benefits when the task was made easier by localising concrete or familiar icons.

## General discussion

Does aesthetic appeal influence performance and, if yes, how might it do so? The current studies examined this question against a small but intriguing backdrop of previous findings suggesting that aesthetic appeal might be more than simply decoration, but could even influence performance, with the possibility that this might be most apparent under challenging conditions.

In two experiments, aesthetic appeal affected task performance only in conditions where the task was inherently difficult—when target icons were complex, abstract or unfamiliar—but not when the task was easy, i.e. when icons were visually simple, concrete or familiar. The current findings clearly do not support a ubiquitous effect of appeal on performance predicted by the ‘prolongation of joyful experience/increased motivation’ hypothesis. When icons were simple, performance was efficient regardless of aesthetic appeal of the stimuli demonstrating that participants were not attempting to prolong their experience with the target icons. Similarly, it was not the case that motivation was increased uniformly when searching for aesthetically appealing stimuli because there were no benefits of appeal for simple icons. However, appeal may have increased motivation for efficient performance when dealing with complex target icons.

Instead, our findings are compatible with the predictions of the ‘positive affect mediation’ hypothesis, which predicts the facilitative effect of stimulus appeal in problem situations. Although previous evidence shows that appeal can increase positive affect (e.g. Hekkert et al., 2003; Kawabata & Zeki, 2004; Leder, Belke, Oeberst, & Augustin 2004), we did not measure affect here and thus it is not possible at present to suggest that the facilitative effect of appeal on performance was indeed mediated via positive affect. Nevertheless, the current results support the general hypothesis that appeal acts to facilitate performance under certain circumstances. Here, those circumstances arose when target localisation was hindered by the target being visually complex, abstract, or unfamiliar. In such trials, appeal facilitated performance, enhancing performance for aesthetically pleasing icons.

The finding that appeal can influence performance echoes research showing that emotion can bias attentional and perceptual systems in such a way as to give processing priority to positive or negative emotion in face processing (e.g. Becker et al., 2011; Fox et al., 2001; LeDoux, 1996; Pratto & John, 1991). The current studies showed that aesthetic appeal can similarly bias attentional and perceptual systems in such a way as to give processing priority to aesthetically appealing stimuli. However, in contrast to the seemingly ubiquitous effect of emotion on face processing, the performance benefits of aesthetic appeal emerged only when the task was difficult (e.g. when target icons were complex, abstract, or unfamiliar). Logically, it seems unlikely that mechanisms purported to have evolved for specialized emotional visual processing of stimuli like faces with high biological or evolutionary relevance, would apply to the aesthetic appeal on performance in a more cognitive domain.

The current studies are the first to show that aesthetic appeal can influence performance in low-level tasks, and where demand characteristics relating to appeal are eliminated. Previous demonstrations of the effect of appeal on performance have been with semantically laden interfaces and using high-level tasks, such as finding information on a website (e.g. Moshagen et al., 2009), or using a mobile phone to send a message (e.g. Moshagen et al., 2009; Sonderegger & Sauer, 2010). The localisation task used here minimises high-level cognitive contributions to performance and allows examination of aesthetic appeal as a factor influencing visual processing. Furthermore, previous work investigating the effects of aesthetic appeal on performance (e.g. Sonderegger & Sauer, 2010; Thüring & Mahkle, 2007), either had participants rate the stimuli for appeal or appeal was the major focus of the task, which would have changed the way participants processed the stimuli. In the current study no mention was made of appeal before, after or during the experiment.

All of the icons used, and their properties, are published (e.g. McDougall et al., 1999) and open to scrutiny and reassessment. Any other variable that might be postulated to underlie the significant effect of appeal on performance shown here would need to be couched within the context of icons and both account for all of the current data as well as stand up to tests of the proposed stimulus dimension. At the current time, aesthetic appeal has proven to have explanatory efficacy above any of the examined dimensions of complexity, concreteness, and familiarity. Although it always remains possible that some unthought of, correlated, stimulus dimension could be affecting performance, any such suggestion needs to first be demonstrated to be plausible within the stimulus set used here.

One future direction of the current work is to examine whether visual appeal can be a feature that guides the deployment of attention. However, although this may be an attractive notion, the evidence so far regarding what constitutes a feature (basic or emergent) in visual search (and thus leads to flat search slopes/pop-out effects) is not encouraging. For instance, despite some evidence showing that faces can influence attentional selection (e.g. Becker et al. 2011; Ohman et al., 2001; Eastwood et al., 2001; Fox et al., 2001; LeDoux, 1996), the balance of evidence is currently against inclusion of faces and facial emotion on the list of features that guide the deployment of attention (e.g. Fox et al., 2000; Nothdurft, 1993; see Wolfe & Horowitz, 2004 for review). Similarly, appeal may lead to more efficient searches but not necessarily to pop-out effects (flat search slopes). Nevertheless, the current findings are important in showing that visual aesthetic appeal boosts performance in a task used in many different real-world settings, where looking for and acting (clicking or other actions) on icons or symbols in an interface is a pervasive activity in our lives.

The current findings are important in the field of human–computer interaction (HCI) because they go beyond the previous evidence suggesting that more aesthetically appealing interfaces are perceived to be more usable (e.g. Kurosu & Kashimura, 1995; Tractinsky et al., 2000), or that appealing interfaces become more usable because users make more of an effort with them (e.g. Wiedenbeck, 1999): they show that appealing interfaces are more usable. This finding is potentially relevant to different types of stimuli and user experience, where optimising performance could have considerable costs. Indeed, people can be sensitive to performance costs as small as 150 ms (e.g. Gray & Boehm-Davis, 2000). Such costs can add up during multi-step interactions with interfaces, which can lead to employing strategies to avoid interfaces in favour of those that maximise efficient performance. This is likely to be particularly important for interfaces, such as websites (e.g. de Wulf, Schillewaert, Muylle & Rangarajan, 2006; Hong & Kim, 2004; Pandir & Knight, 2006; Tarasewich, Daniel & Griffin, 2001; van Schaik & Ling, 2005) and mobile phones (e.g., Sauer & Sonderegger, 2011; Sonderegger & Sauer, 2010).

For icon design, our results suggest that in tasks that require speeded responses, keeping icons visually simple, concrete and familiar, is important for efficient performance. But if icons need to be complex (i.e. in order to convey more complex information), abstract or unavoidably unfamiliar, it is important to invest in designing icons that are as appealing as possible. Indeed, we have shown here that aesthetically enhancing complex, abstract or unfamiliar icons makes them behave more as if they were simple, concrete or familiar, respectively. This is an important finding for complex interfaces often used in time-critical situations such as head-up displays in cockpits and air traffic control.

In conclusion, the current findings show that aesthetic appeal, as a stimulus characteristic, can influence performance in problem situations, such as when the target to be found is complex, abstract or unfamiliar. Future work needs to examine the mechanism with which appeal can influence performance, be it via positive affect or some other emotional or cognitive process.

## Electronic supplementary material

Below is the link to the electronic supplementary material.

ESM 1 (DOCX 240 kb)

## Footnotes

1 Throughout this paper the term aesthetic *appeal* (or just appeal) is used, rather than aesthetic *preference*, since the former refers to the power to attract or arouse interest, while preference refers to selecting one thing over another. Aesthetic appeal here as well in as previous studies (see Reber et al., 2004 for review) refers to mild aesthetic experiences as evidenced via simple judgments by participants made on the basis of liking.

2 Target localization has also been used in visual search tasks, as a more realistic task than the typically used target present or absent task and when a hybrid memory and visual search task is required (e.g. Kunar, Flusberg, & Wolfe, 2008; Wolfe, 2012).

3 We replicated Experiments 1 and 2 using a more diverse sample of participants. Experiment 3 was a replication of Experiment 1 using only male participants aged between 30 and 47 years of age (M = 37.10, SD = 5.56), and Experiment 4 was a replication of Experiment 2 using a sample of 25 male and female participants over 30 years of age (M = 45.77, SD = 11.98). The pattern of results in those two experiments with respect to the interaction between Appeal and Complexity, and between Appeal and Concreteness/Familiarity was identical to those of Experiments 1 and 2 (see Online Supplemental Material).
