# Supplementary material for: When the going gets tough the beautiful get going: aesthetic appeal facilitates task performance
Source: Psychon Bull Rev. 2015 Jan 17;22(5):1243–54. doi: 10.3758/s13423-014-0794-z (PMC4577531; doi:10.3758/s13423-014-0794-z)
Supplement: Supplementary file 1 — (DOCX 240 kb) [file 13423_2014_794_MOESM1_ESM.docx]

**Online Supplementary Material for “When the Going Gets Tough, the Beautiful Get Going: Aesthetic Appeal facilitates Task Performance”**

**Experiment 3**

Experiment 3 examined whether the pattern of findings in Experiment 1 obtained from a sample consisting primarily of University-age female students would be observed in a very different group of participants consisting of male adults over 30.

**Method**

**Participants.** Nineteen male participants aged between 30 and 47 years of age (*M*=37.10, *SD*=5.56) were recruited from staff and students at Swansea University. They received £10 for their participation. They all reported normal or corrected to normal vision and were naïve to purpose of the study.

**Materials, Design and Procedure.** Experimental stimuli and apparatus, design and procedure were identical to that of Experiment 1.

**Results and Discussion**

Error trials (1.08%) were excluded from the analyses of correct RT. There were no differences in error rates between the four icon conditions (all *p*s>.05). Trials with RT greater than 3 seconds were excluded accounting for 1.74% of all correct responses. Correct mean RT per condition is shown in Table 6.

A 2 (Complexity: complex vs. simple) x 2 (Appeal: appealing vs. unappealing) x 9 (Block: 1-9) repeated-measures ANOVA on correct RT revealed no significant three-way interaction, *F* (8, 144)=1.98, *p*>.05, *ε^2^*=.1. The main effect of Complexity was significant, *F* (1, 18)=132.37, *p*<.001, *ε^2^*=.88, with simple icons found faster than complex icons. The main effect of Appeal was not significant, *F* (1, 18)=2.62, *p*>.05, *ε^2^*=.12, but was qualified by a significant Complexity X Appeal interaction, *F* (1, 18)=6.22, *p*=.02, *ε^2^*=.26. Comparisons examining the Complexity X Appeal interaction (Figure 5) showed no significant difference in RT between appealing and unappealing *simple* icons, *t(*18)=.51, *p*>.05. However, *complex* appealing icons were found faster than complex unappealing icons, *t(*18)=2.37, *p*=.03.

*Table 6.* Mean response times (and *standard deviations*) per Complexity and Appeal

condition, across the nine blocks of trials in Experiment 3.

|  | Icon Type | | | | |
| --- | --- | --- | --- | --- | --- |
| Block of trials | Appealing Complex | Appealing Simple | Unappealing Complex | Unappealing Simple | Total |
| Block 1 | 1313.64  (*230.07*) | 1260.65  (*188.49*) | 1394.36  (*241.54*) | 1246.88  (*191.50*) | 1304.39  (*188.97*) |
| Block 2 | 161.62  (*142.51*) | 1061.42  (*123.91*) | 1244.57  (*157.97*) | 1141.23  (*182.98*) | 1176.01  (*131.85*) |
| Block 3 | 1189.32  (*168.64*) | 1074.03  (*137.22*) | 1202.88  (*181.52*) | 1025.59  (*115.05*) | 1122.08  (*132.08*) |
| Block 4 | 1137.64  (*136.11*) | 1025.71  (*145.62*) | 1181.39  (*174.73*) | 1036.39  (*117.57*) | 1096.48  (*109.37*) |
| Block 5 | 1150.71  (*234.01*) | 1020.10  (*135.64*) | 1108.68  (*150.94*) | 1023.35  (*126.72*) | 1069.46  (*132.32*) |
| Block 6 | 1104.30  (*152.82*) | 1057.05  (*155.21*) | 1120.97  (*106.82*) | 1040.39  (*149.14*) | 1084.30  (*114.29*) |
| Block 7 | 1095.83  (*165.85*) | 1014.05  (*135.57*) | 1131.99  (*154.71*) | 1049.29  (*141.42*) | 1077.53  (*124.80*) |
| Block 8 | 1081.34  (*166.43*) | 1000.01  (*142.69*) | 1151.12  (*192.96*) | 972.74  (*118.21*) | 1048.88  (*123,55*) |
| Block 9 | 1055.68  (*174.79*) | 1011.49  (*170.44*) | 1115.12  (*192.96*) | 957.20  (*121.94*) | 1040.50  (*143.41*) |
| Total | 1152.02  (*129.18*) | 1057.74  (*95.25*) | 1180.55  (*125.39*) | 1053.46  (*96.35*) |  |

Finally, there was a significant main effect of Block, *F* (8, 144) = 18.20, *p*<.001, *ε^2^*=.50, with RT reducing in later blocks of trials. Neither the Block X Appeal, *F* (8, 144)=.80, *p*>.05, *ε^2^*=.04, nor the Block X Complexity interaction, *F* (8, 144)=1.41, *p*>.05, *ε^2^*=.07, were significant.


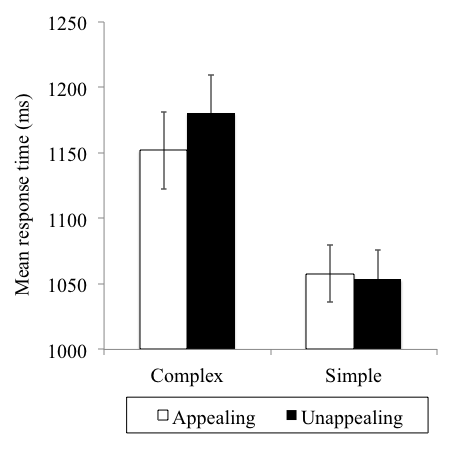


*Figure 5.* Illustration of the Complexity by Appeal interaction in Experiment 3. Error bars represent standard error of the mean.

The pattern of findings in Experiment 3 was identical to that found in Experiment 1, including the significant interaction between Complexity and Appeal. Appeal improved search performance for difficult search in very different samples of participants suggesting the positive effect of aesthetic appeal on visual processing is generalizable.

**Experiment 4**

Experiment 4 was a replication of Experiment 2, recruiting both male and female participants over 30 years of age (since gender did not have an influence on the pattern of results in Experiments 1 and 3).

**Method**

**Participants.** Twenty-five participants took part in this experiment (19 females and 6 males). They were members of the public drawn from the Bournemouth University participant pool or were older volunteers, drawn from students and staff outside the Psychology Department at Bournemouth University. All were over 30 (*M*=45.77, *SD*=11.98), reported normal or corrected vision, and were naïve with respect to the purpose of the experiment.

**Apparatus, Materials, Design and Procedure.**

Stimuli and apparatus, and design and procedure were identical to that of Experiment 2.

**Results and Discussion**

Accuracy was high with errors accounting for 0.02% of trials. As a result, the error data were not considered further. As in Experiment 3, trials with RT greater than 3 seconds accounted for 1.12% of all correct responses and were excluded from the analysis as outliers.

Correct cell mean RT appear in Table 7. A 2 (Concreteness: concrete vs. abstract) x (Appeal: appealing vs. unappealing) x 9 (Block: 1-9) repeated-measures ANOVA on correct mean RT showed no significant three-way interaction, *F* (8,192)=0.67, *p*>.05, *ε^2^*=.03. The main effect of Appeal on RT was significant, *F* (1,24)=10.00, *p*=.004, *ε^2^*=.29, with appealing icons localised faster than unappealing icons. The main effect of Concreteness was also significant, *F* (1,24)=7.92, *p*<.01, *ε^2^*=.25, with concrete icons localised faster than abstract ones. As in Experiment 2, the Concreteness X Appeal interaction (Figure 6A) was significant, *F* (1,24)=28.70, *p*<.0001, *ε^2^*=.54. Pairwise comparisons showed that abstract appealing icons were significantly *faster* than abstract unappealing ones, *t(*24)=5.52, *p*<.0001. In contrast, concrete appealing icons were found *slower* than their unappealing counterparts, *t(*24)=3.06, *p*<.005.

*Table 7:* Mean response time *(*and *standard deviations)* per Concreteness and Appeal condition, across the nine blocks of trials in Experiment 4.

|  | Icon Type | | | |  |
| --- | --- | --- | --- | --- | --- |
| Block of trials | Appealing Abstract | Appealing Concrete | Unappealing Abstract | Unappealing Concrete | Total |
| Block 1 | 1665.50  (*486.15*) | 1676.12  (*446.18*) | 1757.87  (*497.14*) | 1596.07  (*431.89*) | 1673.89  (*454.72*) |
| Block 2 | 1540.85  (*450.65*) | 1586.70  (*450.05*) | 1610.21  *(448.27)* | 1553.11  *(403.52)* | 1572.72  *(421.49)* |
| Block 3 | 1478.33  (*363.27*) | 1566.99  *(440.82)* | 1616.45  *(448.03)* | 1484.13  *(346.60)* | 1539.34  *(3282.90)* |
| Block 4 | 1441.38  *(310.04)* | 1440.85  *(346.97)* | 1627.91  *(445.92)* | 1470.56  *(368.02)* | 1483.25  *(349.63)* |
| Block 5 | 1395.84  *(381.24)* | 1469.44  *(323.42)* | 1491.60  *(397.28)* | 1393.09  *(296.46)* | 1437.49  *(334.75)* |
| Block 6 | 1513.67  *(431.59)* | 1508.23  *(402.36)* | 1612.18  *(419.99)* | 1443.90  *(391.26)* | 1519.50  *(388.55)* |
| Block 7 | 1376.97  *(260.27)* | 1486.38  *(411.71)* | 1551.02  *(453.31)* | 1474.01  (*308.94*) | 1472.10  *(342.66)* |
| Block 8 | 1422.66  *(376.44)* | 1488.00  *(376.44)* | 1509.40  *(340.83)* | 1418.11  *(361.35)* | 1459.54  *(344.73)* |
| Block 9 | 1374.42  *(361.35)* | 1476.28  *(354.62)* | 1502.48  *(354.62)* | 1429.50  *(361.16)* | 1445.67  *(331.40)* |
| Total | 1467.73 *(351.03)* | 1522.11  *(373.33)* | 1582.54  *(395.48)* | 1473.61  *(338.19)* |  |

Finally, there was a significant main effect of Block, *F* (8,192)=13.06, *p*<.0001, *ε^2^*=.35, with concrete icons localised faster than abstract ones in Blocks 1, 4 and 6 [*t*(24)=4.06, *p*<.0001; *t*(24)=2.15, *p*=.04; *t*(24)=2.82, *p*=.009, respectively], while the difference between concrete and abstract icons in the remaining blocks did not reach significance (all *p*s>.05). The Block X Concreteness interaction was also significant, *F* (8,192)=2.12, *p*=.04, *ε^2^*=.08. Bonferroni corrected pairwise comparisons examining this interaction, revealed only a significant difference between concrete and abstract icons (with the former faster than the latter) in Block 1, *t*((24)=4.06, *p*<.005. The Block X Appeal interaction was not significant, *F* (8,192)=1.32, *p*>.05, *ε^2^*=.05.

(A) (B)


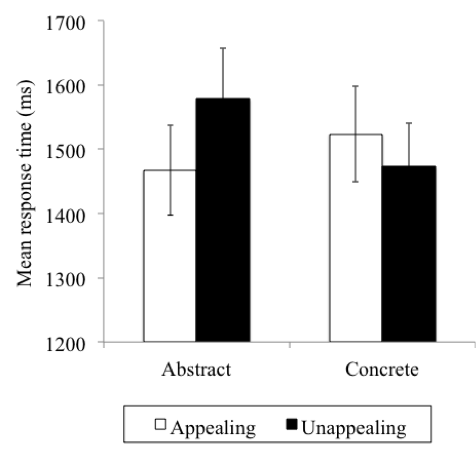

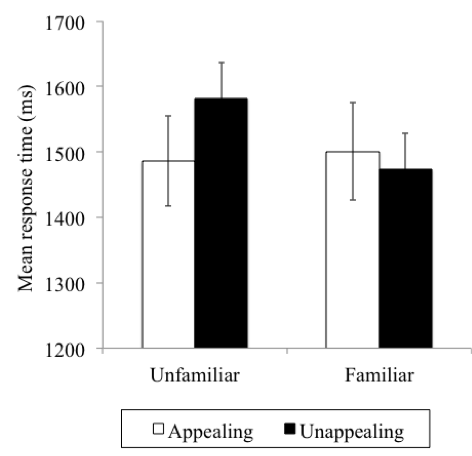


*Figure 6.* Concreteness by Appeal (Panel A) and Familiarity by Appeal (Panel B) interactions in Experiment 4. Error bars represent standard error of the mean.

A second set of analyses was carried out on correct search RT but now the icons were re-coded in terms of Familiarity and Appeal. Correct cell means appear in Table 8. A 2 (Familiarity: familiar vs. unfamiliar) x 2 (Appeal: appealing vs. unappealing) x 9 (Block: 1-9) repeated-measures ANOVA showed no significant three-way interaction, *F* (8,192)<1, *p*>.05, *ε^2^*=.04. All three main effects were significant: Familiarity, *F* (1,24)=22.15, *p*<.001, *ε^2^*=.48; Appeal, *F* (1, 24)=11.01, *p*=.003, *ε^2^*=.31; and Block, *F* (8,192)=12.96, *p*<.001, *ε^2^*= .35. Critically, the Familiarity X Appeal interaction was significant *F* (1,24)=16.80, *p*<.001. The Familiarity X Appeal interaction (Figure 6B) resulted from *faster* localisation RT for appealing unfamiliar icons compared to their unappealing counterparts, *t (*24)=4.64, *p*<.001, while for familiar icons there was no difference between appealing and unappealing icons, *t(*24)=1.67, *p*>.05. Neither the Familiarity X Block nor the Appeal X Block interactions were significant [*F* (8,192)=1.71, *p*>.05, *ε^2^*=.07; Appeal X Block, *F* (8,152)=1.36, *p*>.05, *ε^2^*=.05, respectively].

*Table 8:* Mean response time *(*and *standard deviations)* per Familiarity and Appeal condition, across the nine blocks of trials in Experiment 4.

|  | Icon Type | | | |  |
| --- | --- | --- | --- | --- | --- |
| Block of trials | Appealing Familiar | Appealing Unfamiliar | Unappealing Familiar | Unappealing Unfamiliar | Total |
| Block 1 | 1674.62  (*476.76*) | 1662.54  (*443.10*) | 1596.07  (*431.89*) | 1757.87  (*497.14*) | 1673.89  (*454.71*) |
| Block 2 | 1570.84  (*434.65*) | 1552.19  (*474.36*) | 1553.11  *(402.65)* | 1610.21  *(448.27)* | 1571.86  *(421.00)* |
| Block 3 | 1544.71  (*421.35*) | 1480.44  *(361.98)* | 1484.13  *(347.09)* | 1627.91  *(447.91)* | 1536.45  *(383.10)* |
| Block 4 | 1436.47  *(335.23)* | 1448.17  *(299.27)* | 1470.56  *(368.02)* | 1580.22  *(445.98)* | 1480.77  *(349.26)* |
| Block 5 | 1444.98  *(335.23)* | 1409.09  *(387.13)* | 1393.09  *(297.89)* | 1491.60  *(397.78)* | 1436.98  *(334.22)* |
| Block 6 | 1486.28  *(398.38)* | 1561.34  *(455.85)* | 1443.90  *(386.32)* | 1612.18  *(424.58)* | 1520.77  *(390.09)* |
| Block 7 | 1442.89  *(368.36)* | 1409.18  *(282.34)* | 1447.01  *(309.82)* | 1551.02  (*456.88*) | 1471.31  *(341.42)* |
| Block 8 | 1449.65  *(389.37)* | 1463.76  *(302.67)* | 1418.11  *(366.84)* | 1509.40  *(343.57)* | 1460.31  *(342.58)* |
| Block 9 | 1441.96  *(337.95)* | 1396.72  *(352.52)* | 1429.50  *(356.75)* | 1502.48  *(322.89)* | 1444.69  *(332.37)* |
| Total | 1499.16  *(371.95)* | 1487.05  *(343.58)* | 1473.61  *(338.19)* | 1582.54  *(395.48)* |  |

**Discussion**

The overall pattern of findings for the middle-aged adults, male and female participants in Experiment 4 was very similar to that found in Experiment 2 for younger and predominantly female participants. The critical interaction between Concreteness and Appeal was significant as was the interaction between Familiarity and Appeal. This result confirmed that appeal enhanced performance when performance was made difficult by localizing abstract or unfamiliar targets.

Although both critical interactions – between Concreteness and Appeal and between Familiarity and Appeal – were observed in both Experiments 2 and Experiment 4, there were some differences in the pattern of findings between the two experiments. One difference is that although for the younger participants (Experiment 2) appeal did not make a difference in search RT when the icons were concrete, it took longer for older adults to find appealing concrete icons than their unappealing counterparts. It is not clear why this difference was observed, but one possibility is that participants in Experiment 4 were more responsive to icon concreteness (e.g., Leung, McGrenere, & Graf, 2011). For instance, participants might have attempted to verbalize the icons causing increased latencies. Such verbalization may have been easier for concrete appealing, compared to concrete unappealing icons. Another difference between Experiments 2 and 4 was that in the latter the effects of Concreteness and Familiarity did not reduce as participants gained experience with icons over blocks of trials. In other words, search RT was consistently, across all blocks of trials, faster for concrete and familiar icons. This difference may be due to the greater sensitivity of older adults to icon concreteness, and inevitably, familiarity (e.g., Leung et al., 2011).

Notwithstanding these differences, the pattern of findings regarding the effect of appeal on performance is remarkably similar between Experiments 2 and 4 despite samples differing in age and gender: both for younger and older adults appeal facilitated localization performance under duress.

**References**

Leung, R., McGrenere, J., & Graf, P. (2011). Age-related differences in the initial usability of mobile device icons. *Behaviour & Information Technology*, *30*(5), 629-642.
